# Potential Role of Vitamin D in the Elderly to Resist COVID-19 and to Slow Progression of Parkinson’s Disease

**DOI:** 10.3390/brainsci10050284

**Published:** 2020-05-08

**Authors:** Casey A. Hribar, Peter H. Cobbold, Frank C. Church

**Affiliations:** 1 Department of Pathology and Laboratory Medicine, The University of North Carolina at Chapel Hill School of Medicine, Chapel Hill, NC 27759, USA; casey_hribar@med.unc.edu; 2 Emeritus Professor, Department of Human Anatomy and Cell Biology, University of Liverpool, Liverpool L69 3EG, UK; pcobbold@fireflyuk.net

**Keywords:** Parkinson’s disease, COVID-19, vitamin D, cholecalciferol, therapeutics, antiviral, neurodegeneration, SARS-CoV-2, elderly

## Abstract

While we are still learning more about COVID-19, caused by the novel SARS-CoV-2 virus, finding alternative and already available methods to reduce the risk and severity of the disease is paramount. One such option is vitamin D, in the form of vitamin D_3_ (cholecalciferol) supplementation, due to its potential antiviral properties. It has become apparent that older individuals have a greater risk of developing severe COVID-19, and compared to younger adults, the elderly have lower levels of vitamin D due to a variety of biological and behavioral factors. Older adults are also more likely to be diagnosed with Parkinson’s disease (PD), with advanced age being the single greatest risk factor. In addition to its immune-system-modulating effects, it has been suggested that vitamin D supplementation plays a role in slowing PD progression and improving PD-related quality of life. We completed a review of the literature to determine the relationship between vitamin D, PD, and COVID-19. We concluded that the daily supplementation of 2000–5000 IU/day of vitamin D_3_ in older adults with PD has the potential to slow the progression of PD while also potentially offering additional protection against COVID-19.

## 1. Introduction

### 1.1. COVID-19

In late 2019, a novel coronavirus, originally named 2019-nCoV, began circulating around the world. It was later renamed severe acute respiratory syndrome coronavirus 2 (SARS-CoV-2), after similarities to SARS were noted [1]. The resulting disease caused by SARS-CoV-2 was termed coronavirus disease 2019 (COVID-19) [1]. SARS-CoV-2, like SARS and Middle East Respiratory Syndrome (MERS), is a beta coronavirus that is thought to have originated in bats. 

Like other coronaviruses, SARS-CoV-2 is a non-segmented, positive sense, enveloped, single-stranded RNA virus. SARS-CoV-2 binds to the angiotensin-converting enzyme 2 (ACE2) receptor on type I and type II pneumocytes [1]. Unsurprisingly, the most common symptoms of COVID-19 include fever, cough, and dyspnea, with progression to pneumonia and/or sepsis in more critical cases [2,3]. The most severe cases can lead to marked hypoxemia and a need for mechanical ventilation. This pro-inflammatory state can lead to acute respiratory distress syndrome and cytokine storm syndrome (CSS), likely mediated by a dysregulated immune response involving interleukin-6 (IL-6), tumor necrosis factor alpha (TNF-α), interferon gamma (IFN-γ), interleukin-1 beta (IL-1β), and other inflammatory signaling molecules [4]. 

Current case fatality rates (CFRs) are in flux as we are currently facing this pandemic. Depending on location, estimations range from 1% to 3%, up to 15% or more [1]. While we are still learning more about who is at an increased risk of developing severe COVID-19, two groups have been commonly identified as having worse outcomes: people of advanced age and those with pre-existing conditions (or both). Currently, there is no scientific consensus on a cure for COVID-19; however, several vaccines are in development, along with multiple clinical trials surrounding medications currently on the market, such as remdesivir and hydroxychloroquine [1]. In the interim, other commonly available, potentially immune-impacting options have been postulated to provide protective effects or reduce the severity of COVID-19. One of these theorized options is vitamin D. 

### 1.2. Vitamin D: Special Considerations in the Elderly

Vitamin D and its activation are intertwined with the action of parathyroid hormone (PTH), as well as the maintenance of calcium and phosphate balance [5,6,7]. Vitamin D is introduced into the body in two ways: through sunlight and through diet or supplementation [5,6,7]. In the skin, vitamin D precursor 7-dehydrocholesterol is activated through UVB rays where it becomes vitamin D_3_. Vitamins D_2_ and D_3_ can be obtained directly from supplementation and/or fatty fish and fortified milks, cereals, juices, and dairy products in the diet. Vitamin D_2_ and D_3_ are then converted in the liver to 25-hydroxyvitamin D (25(OH)D_3_), which circulates in the serum bound to vitamin D binding proteins (DBPs). Of note, 25(OH)D_3_ in the serum is the best marker for determining vitamin D deficiency. This form of vitamin D becomes activated in kidneys by 25-hydroxyvitamin D-1-alpha-hydroxylase (1-OHase, induced by PTH), and becomes 1,25-dihydroxyvitamin D (1,25(OH)_2_D, Calcitriol) [8,9]. Low levels of vitamin D are more common amongst older or elderly individuals. This may be due to lessened mobility and time outside in the sunlight, increased adiposity, reduced rates of synthesis of vitamin D in the skin, reduced appetite, and reduced vitamin D absorption in the gut [8,9].

Due to the widespread distribution of the vitamin D receptor (VDR) throughout the body, it has been postulated that it plays a role in many critical functions as well as pathologies. Vitamin D deficiency or dysregulation has been associated with osteoporosis, diabetes, immune dysregulation, inflammation, certain malignancies, hypertension, cardiovascular disease, and cognitive decline, among other maladies [8,9]. Interestingly, most of these pathologies are also associated with increasing age. The direction of causality in these relationships, if there is one, is yet to be determined. However, several studies have suggested that achieving and maintaining adequate vitamin D levels may improve clinical outcomes from common diseases and/or help reduce the risk of their development [8,9,10].

### 1.3. Parkinson’s Disease: The Intersection of COVID-19 and Vitamin D Deficiency

It has also been suggested that neurological/degenerative, motor, and cognitive issues are impacted by vitamin D, with lower levels potentially increasing the risk and adverse effects of these disorders [8,9,10,11,12]. One such condition that may be impacted by vitamin D levels is Parkinson’s disease (PD). PD is a neurodegenerative disorder marked by neuronal cell death in the pars compacta region of the substantia nigra [13,14,15,16]. This reduces the ability to synthesize dopamine and leads to the common hallmarks of PD: tremor, postural instability, bradykinesia, and rigidity. Additional associated symptoms of PD include depression, sleep disruptions, and bowel or bladder dysfunction [13]. The most common treatment regimens for PD aim to increase dopamine levels [17,18,19,20,21,22,23]; however, some patients also find benefit in exercise [24,25,26,27,28] and in complementary and alternative medicine methods [29,30]. PD is typically a disease of older individuals, with increasing age being the greatest risk factor for developing the condition [13,14].

With the current COVID-19 pandemic [31], finding alternative, already available methods to reduce the risk and severity of the disease is paramount. In this article, we consider if vitamin D deficiency in the elderly, especially those with PD, contributes to an increased susceptibility to COVID-19. We also discuss whether vitamin D supplementation might provide a means of increasing protection from COVID-19 while helping to slow PD progression and improve quality of life.

## 2. Advancing Our Understanding of Viral Diseases and Parkinson’s

### 2.1. Possible Antiviral Action of Vitamin D

Previous studies have found mixed results when it comes to vitamin D and its impact on illness, specifically respiratory tract illnesses like influenza [10,32,33,34]. The current landscape is dominated by smaller studies with specific populations, and is lacking in numerous, large-scale, randomized controlled trials (RCTs). This may be why a consensus on vitamin D’s antiviral actions has not been found at this time. However, several properties of vitamin D, and results from animal and human in vitro and in vivo studies, suggest that antiviral benefits are not out of the question [8,32,33,35,36,37,38,39,40,41,42].

Vitamin D plays a role in the innate immune system in a variety of ways. In conjunction with toll-like receptors (TLRs), activated vitamin D increases the expression of cathelicidin and human beta defensin 2 peptides [8,32,33,35,36,38]. Cathelicidin (LL-37) interferes with bacterial cell membranes. This property is thought to extend to viruses as well, particularly enveloped viruses, and may impact viral entry [32,35]. Lung epithelial cells have high levels of 1-OHase, allowing for increased cathelicidin in the respiratory tract and potential protection against respiratory illnesses. Human beta defensin 2 can serve as a chemoattractant for other inflammatory cells [8,32,33,35,36,38]. Vitamin D may also increase capillary permeability to help deliver inflammatory mediators to the site of infection [35]. Further, it is thought to play a role in the maintenance of a variety of cell junction types [10,34]. Strong physical barriers through effective cell junctions is the body’s first line of defense against pathogens. Vitamin D and the activation of its receptors also induces invariant natural killer (NK) T cells, forming a bridge to the adaptive immune response [33].

From an adaptive immune system perspective, the binding of VDRs by activated vitamin D leads to changes in gene transcription. Specifically, vitamin D leads to a blunting of the Th1 immune response, and favors Th2 and regulatory T cell responses [32,33,36]. This leads to a decrease in pro-inflammatory cytokines associated with the Th1 response, such as IL-6, TNF-α, and IFN-γ, and an increase in anti-inflammatory cytokines associated with the Th2 immune response, such as IL-10 and IL-2 [8,33,36]. The Th2 response also serves to further dampen the Th1 response, while the Treg response further reduces inflammation. Many illnesses, including COVID-19, have the potential to lead to immune system dysregulation and cytokine storm, often involving IL-6, TNF-α, and IFN-γ [4]. By downregulating pro-inflammatory cytokines and upregulating anti-inflammatory cytokines, vitamin D may be capable of preventing this severe complication related to COVID-19 and other viral illnesses. Although more data are needed to understand the cytokine regulatory abilities of vitamin D, some suggest that these benefits may be most pronounced with longer-term vitamin D supplementation, rather than large, individual bolus doses [8]. Long-term treatment with lower doses of vitamin D may also reduce the risk of vitamin D-related toxicities [33].

Vitamin D may also have antioxidant properties as well as the ability to increase telomere length and DNA stability [8,9,10]. Further, vitamin D has been theorized to help bolster immune responses to vaccination, a factor that may be critical to consider when vaccines are eventually developed against COVID-19 [33]. As mentioned, past studies have had mixed results when it comes to vitamin D’s antiviral properties, but some have found reductions in illness development, duration, and severity in relation to adequate or enhanced vitamin D levels [8,33,36]. Specific viruses considered in some of these efforts included influenza, chronic hepatitis B, dengue, HSV-1, bacterial and viral pneumonia, RSV, and rotavirus, among others [36,37,39]. Evidence of vitamin D’s impact on the ACE2 receptor is conflicting. Several suggest that vitamin D and its receptor may directly down-regulate the ACE2 receptor, thus, decreasing the risk of infection with COVID-19 [43,44,45,46]. By contrast, others suggest that vitamin D up-regulates ACE2 [47,48]. While this may play a role in helping to mitigate the later effects of COVID-19, it may lead to an increased infection risk. More research is needed on the relationship between vitamin D and the ACE2 receptor, and how this may impact COVID-19 risk and pathogenesis.

### 2.2. Possible Slowing of PD Progression

Beyond its potential antiviral properties, vitamin D may play a role in PD development and progression. Several studies have suggested that patients with PD, especially earlier stage PD, have lower baseline levels of 25(OH)D_3_ than healthy controls, and that lower levels of 25(OH)D_3_ correspond with an increased disease prevalence and severity [11,12,38]. It is unclear if or why vitamin D, or its lack thereof, may play a role in PD development and progression, but some have noted that VDRs are located on dopaminergic neurons in the substantia nigra, an area degenerated in PD [11,38]. Interestingly, VDRs have super-promoter activity for the oxidative stress pathway (notably, Nrf2-KEAP), which directly promotes the production of antioxidants as well as calcium pumps and channels [49]. The disruption of these cellular signals and circuits from vitamin D deficiency has been implicated as a cause of idiopathic PD [49]. It has been postulated that supplementing vitamin D, especially for individuals with lower baseline levels, may protect dopaminergic neurons and their receptors. Animal models have demonstrated this neuroprotective possibility [38]. To our knowledge, there are no completed or ongoing clinical trials on vitamin D’s potential to protect these neurons in humans.

Vitamin D’s impact may be pervasive across all aspects of PD, including motor and non-motor symptoms. For example, Sleeman et al., concluded that variance in motor impairment severity at 36 months was predicted by age, dosage of dopaminergic medications, motor score, and baseline serum 25(OH)D_3_ levels, with lower D_3_ being associated with worsened progression [38]. Additionally, in individuals with PD who have impaired motor functioning, postural instability, and balance difficulties, falls may be more common. Low vitamin D impairs calcium homeostasis, leading to osteoporosis and an increased risk of bone fractures or worsened outcomes from a fall [9,38]. A significant fall or fracture can greatly reduce quality of life for a patient with PD. 

Further, Peterson et al. concluded that, among PD patients without significant dementia, stronger performances on neuropsychiatric tests were associated with higher 25(OH)D_3_ levels in the blood. This was especially true for verbal fluency and verbal memory [12]. This group also concluded that vitamin D may play a role in reducing depression [12]. Utilizing vitamin D to improve bone health, reduce the risk of serious injury from falls, improve cognition and memory, and improve mood may lead to increased quality of life and slowed disease progression for PD patients.

### 2.3. Safety and Adverse Events of Vitamin D Supplementation

Although it is a fat-soluble vitamin, it is rare to experience an overdose of vitamin D. Vitamin D overdoses related to sunlight exposure almost never occur. Hypervitaminosis D is almost exclusively related to over supplementation [9]. It has been postulated that direct synthetic analogs of 25(OH)D_3_ do not bind as well to DBPs, making oral D_2_ or D_3_ (cholecalciferol) supplementation the preferred option for increasing vitamin D [8]. The United States Institute of Medicine recommended a conservative daily vitamin D limit of 4000 IU/day; however, safe daily supplementation may reach closer to 7500–10,000 IU/day [36]. Extremely large bolus doses may be utilized in dire clinical scenarios, but only when under the direct supervision of a physician, since the risk of overdose and adverse effects increases with these approaches. 

Those who have investigated vitamin D’s impact on the immune response suggest that a serum level of 40–60 ng/mL (100–150 nmol/L) 25(OH)D_3_ may be necessary for respiratory infection prevention [36]. This is in contrast to the roughly 30 ng/mL recommended for skeletal health and cognitive function, and 50–80 ng/mL for the prevention of other chronic conditions, including hypertension and cardiovascular disease [8,9]. The adverse effects of vitamin D_3_ supplementation are generally not experienced until serum concentrations of 25(OH)D_3_ exceed 150 nmol/L [9]. The most common side effects experienced are poor appetite, nausea, vomiting, constipation, weakness, or weight loss. Excessive vitamin D_3_ supplementation may lead to increases in calcium, leading to disorientation, arrhythmias, confusion, fatigue, and gastrointestinal upset. Excess calcium may also lead to nephrolithiasis and kidney damage [8,9]. Despite these risks, the hypervitaminosis of vitamin D is rare, and would require excessive daily D_3_ supplementation for an extended period of time, making D_3_ a safe supplement in most populations.

## 3. Fitting the Pieces of the Puzzle Together

**Figure 1:**
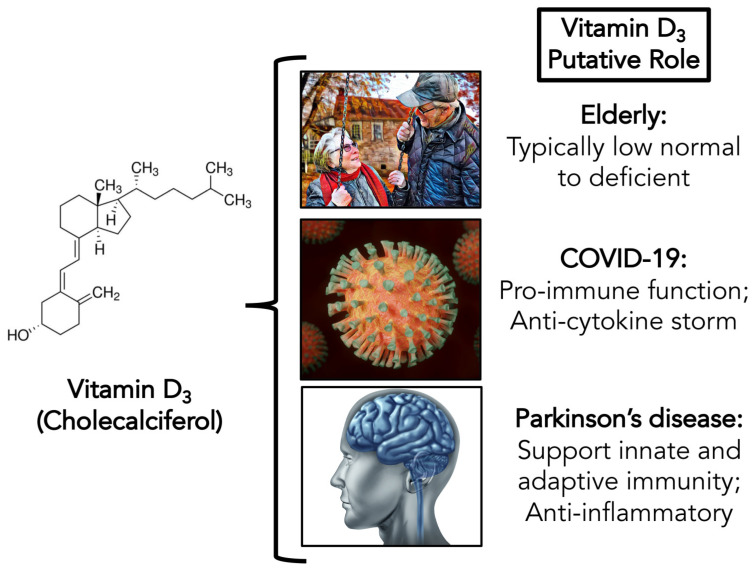
The potential roles of vitamin D_3_ supplementation in the elderly, with special consideration to those with Parkinson’s disease and at risk of developing COVID-19.

Although more RCTs are needed, current literature suggests that vitamin D levels can impact the immune system and may impact the risk of developing common medical conditions. Vitamin D may also impact the severity of these conditions as well. Those with low vitamin D levels may be at a higher risk of developing infections, including respiratory infections, due to immune system dysregulation. An increased risk of comorbid conditions as a result of low vitamin D levels may also lead to worsened outcomes from infections, including COVID-19. Vitamin D levels are lower in elderly individuals, a group that is also at higher risk of COVID-19, as shown in Figure 1. 

Individuals with PD are typically older and have been independently noted to have lower vitamin D levels than their healthy counterparts. This places them at a unique junction in which their risk of developing COVID-19 may be increased more so than that of the general elderly population. Furthermore, evidence has suggested that vitamin D supplementation may be beneficial in improving both motor and non-motor symptoms of PD, and decreasing the risk of bone fractures after a fall. All of these factors may contribute to an increase in quality of life for patients with PD. 

By combining our knowledge of the antiviral properties of vitamin D with our understanding of vitamin D’s impact on PD-related quality of life, it is logical to recommend vitamin D_3_ supplementation to this population for both improvements in PD progression and potential COVID-19-related benefits, as shown in Figure 1. Vitamin D supplementation is best achieved through D_3_ (cholecalciferol) supplements, which are widely available and relatively inexpensive. The favorable safety profile of D_3_ supplements also makes them an ideal choice for those deficient in vitamin D. We suggest a daily dosage of 2000–5000 IU/day of vitamin D_3_; however, higher doses may be needed for those with severe deficiency or in extraordinary situations where other clinical options are limited. Due to the ongoing benefits this may provide for people with PD, we recommend continuing this supplementation for as long as possible; potentially life-long, if feasible. The ideal 25(OH)D_3_ concentration should be around 40–60 ng/mL, the physiological level [50], and routine serum monitoring may be beneficial to determine the lowest daily D_3_ dosage needed for optimal benefit. Longer-term supplementation is recommended rather than individual, large bolus doses, unless an initial bolus is required in severe deficiencies. Maintaining adequate vitamin D levels may also help improve immune responses to a COVID-19 vaccination when one becomes available, further reducing risk. 

## 4. Conclusions

Vitamin D may have antiviral properties and play a role in protecting against infections, including respiratory illnesses. Elderly individuals are generally deficient in vitamin D, and people with PD are even more likely to be deficient. Supplementation with vitamin D_3_ may help improve the motor and non-motor symptoms of PD, thus improving quality of life. Although further study is needed, daily supplementation with 2000–5000 IU/day of vitamin D_3_ in individuals with PD may be beneficial in reducing the risk and severity of COVID-19.
